# BMC Anesthesiology reviewer acknowledgement, 2014

**DOI:** 10.1186/1471-2253-15-10

**Published:** 2015-02-06

**Authors:** Thomas A Rowles

**Affiliations:** BioMed Central, Floor 6, 236 Gray’s Inn Road, London, WC1X 8HB UK

## Abstract

The editors of BMC Anesthesiology would like to thank all our reviewers who have contributed their time to the journal in Volume 14 (2014).

**Mohamed Abdulatif**

Egypt

**Serefden Acikgoz**

Turkey

**Yushi Adachi**

Japan

**M. Christopher Adams**

USA

**Massimo Agostoni**

Italy

**Vassilis Aidinis**

Greece

**Ibrahim Ozkan Akinci**

Turkey

**Daniela Alampi**

Italy

**Giancarlo Albo**

Italy

**Charles Algert**

Australia

**Nicole Almenrader**

Italy

**Michael Andrawes**

USA

**Christoph Ansorge**

Sweden

**Richard Applegate**

USA

**Karim Asehnoune**

France

**Eftihia Asprodini**

Greece

**Aimee Aysenne**

USA

**Aranya Bagchi**

USA

**Michael Barrington**

Australia

**Marc Beaussier**

France

**Helene Beloeil**

France

**Jan Belohlavek**

Czech Republic

**Dan Benhamou**

France

**Monika Berns**

Germany

**Andrew Bersten**

Australia

**Azize Bestas**

Turkey

**Indiradevi Bhagavatula**

India

**Gianni Biancofiore**

Italy

**Daniele Biasucci**

Italy

**Elena Bignami**

Italy

**Jan Blaha**

Czech Republic

**Kevin Blaine**

USA

**Maria Boddi**

Italy

**Manuela Bonizzoli**

Italy

**Giovanni Borghi**

Italy

**Marie Bosman**

United Kingdom

**Alex Bottle**

United Kingdom

**Pervin Bozkurt Sutas**

Turkey

**Barbara Brandom**

USA

**Nicola Brienza**

Italy

**Yuriy Bronshteyn**

USA

**Bruno Buchholz**

Argentina

**Eric Busch**

USA

**John Butterworth**

USA

**Elif Cadirci**

Turkey

**Brian Cairns**

Canada

**Guy Cammu**

Belgium

**Anna Camporesi**

Italy

**Gianluca Cappelleri**

Italy

**Cesar Carcamo**

Chile

**Maria Carmona**

Brazil

**Daniel Carr**

USA

**Michele Carron**

Italy

**Davide Cattano**

USA

**Erol Cavus**

Germany

**Hulya Celebi**

Turkey

**Maurizio Cereda**

USA

**Jonathan Charnin**

USA

**Cosimo Chelazzi**

Italy

**Catherine Chen**

USA

**Vidya Chidambaran**

USA

**Daniel Chipman**

USA

**Stephen Choi**

Canada

**Christopher Choukalas**

USA

**Gilda Cinnella**

Italy

**Yann-Erick Claessens**

Monaco

**Nathalie Clavier**

France

**Richard Cooper**

Canada

**Menino Osbert Cotta**

Australia

**Gregory Crosby**

USA

**Giovanni Cucchiaro**

USA

**Douglas Curran-Everett**

USA

**J. Randall Curtis**

USA

**Wojciech Dabrowski**

Poland

**Aniello De Nicola**

Italy

**Gennaro De Pascale**

Italy

**Nicolas De Prost**

France

**Francesco Giuseppe De Rosa**

Italy

**Assunta De Vitis**

Italy

**Giorgio Della Rocca**

Italy

**Joseph Deng**

USA

**Mark Dershwitz**

USA

**Andre Dewolf**

USA

**Franklin Dexter**

USA

**Matteo Di Nardo**

Italy

**Pierre Diemunsch**

France

**Yalim Dikmen**

Turkey

**Dawn Dillman**

USA

**Ning Ding**

China

**Sang-Hwan Do**

South Korea

**Marie-Agnès Docquier**

Belgium

**William Donaldson**

United Kingdom

**Anne Donovan**

USA

**Roberto Dossi**

Italy

**Michael Duggan**

USA

**Roderic Eckenhoff**

USA

**Richard Egan**

United Kingdom

**Matthias Eikermann**

USA

**Douglas Eleveld**

Netherlands

**Mohammad Elorbany**

USA

**Ali Elsayes**

USA

**Mohamed El-Tahan**

Egypt

**Emre Erbabacan**

Turkey

**Ali Fuat Erdem**

Turkey

**Nauder Faraday**

USA

**Ehab Farag**

USA

**David Faraoni**

Belgium

**Hassan Farhan**

USA

**Argyro Fassoulaki**

Greece

**John Feiner**

USA

**Paolo Feltracco**

Italy

**Karim Fikry**

USA

**Ryan Fink**

USA

**Alfonso Fiorelli**

Italy

**Michael Firstenberg**

USA

**Stuart Forman**

USA

**Gyorgy Frendl**

USA

**Inez Frerichs**

Germany

**Eva Fuentes**

USA

**Tatsuma Fukuda**

Japan

**Tibor Fulop**

USA

**Richard Galgon**

USA

**Samuel Galvagno**

USA

**Marcelo Gama De Abreu**

Germany

**Roser Garcia-Guasch**

Spain

**Marc Garnier**

France

**Lene Heise Garvey**

Denmark

**Mona Ring Gätke**

Denmark

**Markus Gehling**

Germany

**Marco Gemma**

Italy

**Marc Gentili**

France

**Mohamed Amin Ghobadifar**

Iran

**Chiara Giorni**

Italy

**Massimiliano Greco**

Italy

**Vadim Gudzenko**

USA

**Jean Guglielminotti**

France

**Freedom Nkhululeko Gumedze**

South Africa

**Dhanesh Gupta**

USA

**Harshad Gurnaney**

USA

**Thomas Hachenberg**

Germany

**Robert G Hahn**

Sweden

**Seetharaman Hariharan**

Trinidad and Tobago

**Erik K. Hartmann**

Germany

**Soshi Hashimoto**

Japan

**Randolph Hastings**

USA

**Goran Hedenstierna**

Sweden

**Jan Hendrickx**

Belgium

**Matthias Heringlake**

Germany

**Ross Hofmeyr**

South Africa

**Masaru Honda**

Japan

**Jean-Louis Horn**

USA

**Hisham Hosny**

Egypt

**Tim Howes**

United Kingdom

**Martin Huelsmann**

Austria

**Jennifer Hunter**

United Kingdom

**Jung-Won Hwang**

South Korea

**Luiz Eduardo Imbelloni**

Brazil

**Craig Jabaley**

USA

**Narasimhan Jagannathan**

USA

**Michael James**

South Africa

**Wei Jiang**

China

**Daniel Johnson**

USA

**Girish Joshi**

USA

**Rebecca Kalman**

USA

**Dharshi Karalapillai**

Australia

**Menelaos Karanikolas**

USA

**Brad Karon**

USA

**Zbigniew Karwacki**

Poland

**Marc Kastrup**

Germany

**Joel Katz**

Canada

**Nermin Kelebek Girgin**

Turkey

**Max Kelz**

USA

**Hanan Khafagy**

Egypt

**Bodin Khwannimit**

Thailand

**Rainer Kiefmann**

Germany

**Hae-Keum Kil**

South Korea

**Chol Kim**

Japan

**Ji Young Kim**

South Korea

**Jong Yeop Kim**

South Korea

**Kristopher Kimmell**

USA

**Detlef Kindgen-Milles**

Germany

**Michel Kindo**

France

**Erik Kistler**

USA

**Kemalettin Koltka**

Turkey

**Ozlem Korkmaz Dilmen**

Turkey

**Irene Kourbeti**

Greece

**Morten Tange Kristensen**

Denmark

**Michael Alexander Kuiper**

Netherlands

**Eiichi Kumamoto**

Japan

**Alexander Kuo**

USA

**John Laffey**

Canada

**Giovanni Landoni**

Italy

**Meghan Lane-Fall**

USA

**Malinee Laopaiboon**

Thailand

**Asad Latif**

USA

**Bruno Laviolle**

France

**J. Adam Law**

Canada

**Chiara Lazzeri**

Italy

**Jae W Lee**

USA

**Po-Shun Lee**

USA

**Sangseok Lee**

South Korea

**Christian Lehmann**

Canada

**Hendrikus Lemmens**

USA

**Edmund Leung**

United Kingdom

**Vincent Lew**

USA

**Kent Lewandrowski**

USA

**Hsin Jung Lin**

USA

**Jun Lin**

USA

**Benigno Linares Segovia**

Mexico

**Michael Lipnick**

USA

**Steven Lisco**

USA

**Celia Lloret-Linares**

France

**Anne Lui**

Canada

**Nadia Lunardi**

USA

**Carl Lynch**

USA

**Christopher Lysakowski**

Switzerland

**Jean-Marc Malinovsky**

France

**Laxmaiah Manchikanti**

USA

**Jeff Mandel**

USA

**Dauri Mario**

Italy

**Claes-Roland Martling**

Sweden

**Christie Mato**

Nigeria

**Eckhard Mauermann**

Switzerland

**Tommaso Mauri**

Italy

**Courtney Maxey-Jones**

USA

**Lynne Maxwell**

USA

**Michael Mazzeffi**

USA

**Colin Mccartney**

Canada

**Duncan Mclean**

USA

**Graeme Mcleod**

United Kingdom

**Kamal Medlej**

Lebanon

**Francesco Menichetti**

Italy

**Guniz Meyanci Koksal**

Turkey

**Pavel Michalek**

United Kingdom

**Cyrus Mintz**

USA

**Calin Mitre**

Romania

**Nicholas Mohr**

USA

**Hasse Møller-Sørensen**

Denmark

**Zsolt Molnar**

Hungary

**Andrea Montisci**

Italy

**Ingrid Moreno Duarte**

USA

**Rory Morty**

Germany

**Jeremi Mountjoy**

USA

**George Mychaskiw**

USA

**Yasuko Nagasaka**

USA

**Bala Nair**

USA

**Giuseppe Natalini**

Italy

**Vladimir Nekhendzy**

USA

**Kelly Newman**

USA

**Pornswan Ngamprasertwong**

USA

**Warwick Ngan Kee**

China

**Albert Nguyen**

USA

**David Nicolau**

USA

**Kamilla Nielsen**

Denmark

**Diederik Nieuwenhuijs**

Netherlands

**Catherine Nix**

Ireland

**Marko Noc**

Slovenia

**Anders Kehlet Nørskov**

Denmark

**Mohamed Nour**

USA

**Ala Nozari**

USA

**Omar Omar**

USA

**Andrea Orfanakis**

USA

**Sisse R Ostrowski**

Denmark

**Megan Othus**

USA

**Yen-Chuan Ou**

Taiwan

**Mehmet Ozcan**

USA

**Dilek Ozcengiz**

Turkey

**Peter Paal**

Austria

**Sher-Lu Pai**

USA

**Krishna Parekh**

USA

**Martyn Parker**

United Kingdom

**Pisano Pascale**

France

**Maurizio Passariello**

Italy

**Amit Patel**

USA

**Kashyap Patel**

Australia

**Piyush Patel**

USA

**Vittorio Pavoni**

Italy

**Jason Peart**

Australia

**Paolo Pelosi**

Italy

**Yingwei Peng**

Canada

**Pernille Lykke Petersen**

Denmark

**Tobias Piegeler**

Switzerland

**Marina Pieri**

Italy

**Annop Piriyapatsom**

USA

**Massimiliano Pirrone**

Italy

**Jean-Francois Pittet**

USA

**Valentina Porta**

Brazil

**Alen Protic**

Croatia

**Prakash P Punjabi**

United Kingdom

**Lorenzo Quario Rondo**

Italy

**Douglas Raines**

USA

**Saifudin Rashiq**

Canada

**Lars Rasmussen**

Denmark

**Kristofer Rau**

USA

**James Rhee**

USA

**Paul Riegelhaupt**

USA

**Hynek Riha**

Czech Republic

**Joseph Rinehart**

USA

**Katherine Robinson**

Australia

**Antonio Rodríguez Núñez**

Spain

**Dae-Hyun Roh**

South Korea

**Stefano Romagnoli**

Italy

**Carl Rosow**

USA

**Kenneth Rothfield**

USA

**Gabriel Rusanescu**

USA

**Alisdair Ryding**

United Kingdom

**Jung-Hee Ryu**

South Korea

**Mohammad Ali Sahmeddini**

Iran

**Sam Salman**

Australia

**Adam Sapirstein**

USA

**Jana Sawynok**

Canada

**Thomas Scheeren**

Netherlands

**Christoph Schlimp**

Austria

**Andre Schmidt**

Brazil

**Ulrich Schmidt**

USA

**Andreas Schneider**

Germany

**Tobias Schuerholz**

Germany

**Stephan Schug**

Australia

**Peter Schulman**

USA

**Marcus J Schultz**

Netherlands

**Kurt Schumacher**

USA

**Wolfram Schummer**

Germany

**Martin Schuster**

Germany

**Lalit Sehgal**

India

**Nuzhet Mert Senturk**

Turkey

**Ary Serpa Neto**

Netherlands

**Michael Serpell**

United Kingdom

**You Shang**

China

**Reza Shariat Moharari**

Iran

**Kenneth Shelton**

USA

**Shiqian Shen**

USA

**Koh Shingu**

Japan

**Matthew Sigakis**

USA

**Paolo Silvani**

Italy

**Brett Simon**

USA

**David Simpson**

USA

**Aparna Sinha**

India

**Ashish Sinha**

USA

**Sarath Sistla**

India

**Markus Skrifvars**

Finland

**Andreas Skyschally**

Germany

**Callum Sleigh**

New Zealand

**Anja Slikkerveer**

Netherlands

**Mieke Soens**

USA

**Sohan Solanki**

India

**Xue-Jun Song**

USA

**Massimiliano Sorbello**

Italy

**Philip Spencer**

USA

**Peter Spieth**

Germany

**Vanessa Stadlbauer**

Austria

**David Stahl**

USA

**Joel Starkopf**

Estonia

**Stanislaw Stawicki**

USA

**Bernd Stegmayr**

Sweden

**Kenan Stern**

USA

**Francesca Stoppa**

Italy

**Bala Subramaniam**

USA

**Rajeev Subramanyam**

USA

**Takahiro Suzuki**

Japan

**Andrea Székely**

Hungary

**Szilard Szucs**

Ireland

**Paul Szumita**

USA

**Christopher Tainter**

USA

**Boyd Taylor Thompson**

USA

**Anders Thorell**

Sweden

**Kevin Thornton**

USA

**Yuke Tian**

China

**Kevin Tremper**

USA

**Tanja Treschan**

Germany

**Luigi Tritapepe**

Italy

**Melek Tulunay**

Turkey

**Andrew Udy**

Australia

**Basak Ugurlu**

Turkey

**Susana Vacas**

USA

**Tom Van Der Poll**

Netherlands

**Pieter Van Der Starre**

USA

**Arthur Van Zanten**

Netherlands

**Atul Vats**

USA

**Nicholas Ventham**

United Kingdom

**Marcel Vercauteren**

Belgium

**Tomas Vymazal**

Czech Republic

**Alexander Wahba**

Norway

**Samuel Wald**

USA

**Fuzhou Wang**

China

**Marcin Wasowicz**

Canada

**Timothy Webb**

USA

**Joseph Weidman**

USA

**Avi Weinbroum**

Israel

**Toby Weingarten**

USA

**Nicolas Weiss**

France

**Tyler Wellman**

USA

**Elizabeth Whitlock**

USA

**Christoph Wiese**

Germany

**Craig Williamson**

USA

**David Williamson**

Canada

**Cynthia Wong**

USA

**Hector Wong**

USA

**Hermann Wrigge**

Germany

**Hinnerk Wulf**

Germany

**Christian Wunder**

Germany

**Hannah Wunsch**

USA

**Emre Yalcinkaya**

Turkey

**Bin Yang**

United Kingdom

**Daniel Dante Yeh**

USA

**Jun Rho Yoon**

South Korea

**Nayer Youssef**

Canada

**Ahmed Zaafran**

USA

**Massimo Zambon**

Italy

**Alberto Zanella**

Italy

**Christine Zanghi**

USA

**Emanuela Zannin**

Italy

**Valerie Zaphiratos**

Canada

**Nick Zavras**

Greece

**Fatih Zor**

Turkey

**Martin Zoremba**

Germany

